# Effect of Egg Washing and Correlation between Eggshell Characteristics and Egg Penetration by Various *Salmonella* Typhimurium Strains

**DOI:** 10.1371/journal.pone.0090987

**Published:** 2014-03-12

**Authors:** Vaibhav C. Gole, Kapil K. Chousalkar, Juliet R. Roberts, Margaret Sexton, Damian May, Jessica Tan, Andreas Kiermeier

**Affiliations:** 1 School of Animal and Veterinary Sciences, The University of Adelaide, Roseworthy, SA, Australia; 2 School of Environmental & Rural Science, The University of New England, Armidale, NSW, Australia; 3 Primary Industries and Regions SA, Adelaide, SA, Australia; 4 South Australian Research and Development Institute, Adelaide, SA, Australia; Institut National de la Recherche Agronomique, France

## Abstract

*Salmonella* is an important foodborne pathogen, causing an estimated 11,992 cases of infection in Australia per year. Egg or egg product related salmonellosis is a major concern for the egg industry. Worldwide, *S.* Typhimurium is one of the most common serovars identified in *Salmonella* food poisoning cases. The current study investigated the ability of five *S.* Typhimurium strains to penetrate washed and unwashed eggs using whole egg and agar egg penetration methods. All *S.* Typhimurium strains were able to penetrate eggshells and survive in egg albumen (at 20°C) according to whole egg penetration results. Polymerase Chain Reaction results demonstrated that *S.* Typhimurium strain 2 (10^3^ and 10^5^ CFU/mL), and strain 5 (10^3^ and 10^5^ CFU/mL) egg penetration was significantly higher (p<0.05) in washed eggs when compared to unwashed eggs. Statistical analysis of the agar penetration experiment indicated that *S.* Typhimurium was able to penetrate washed eggs at a significantly higher rate when compared to unwashed eggs (p<0.05). When compared to unwashed eggs, washed eggs also had significantly damaged cuticles. Statistical analysis also indicated that eggshell penetration by *S.* Typhimurium was related to various eggshell ultrastructural features such as cap quality, alignment, erosion, confluence, Type B bodies and cuticle cover.

## Introduction

*Salmonella* spp. have been one of the most important food poisoning pathogens throughout the last century and remain a challenge for microbiology and public health [1]. It is estimated that 1.3 billion incidences of nontyphoidal salmonellosis occur throughout the world annually [2]. The annual report of the OzFoodnet network [3] reported 11,992 cases of *Salmonella* infection in Australia in 2010, with an estimated annual cost due to all food borne illness of $1.2 billion [4]., Eggs are often implicated in the cases of food poisoning due to salmonellosis [3], which can be acquired by the ingestion of raw or undercooked eggs. Intact eggs can be contaminated by *Salmonella* using two possible routes: vertical transmission and horizontal transmission. Vertical transmission occurs as a result of *Salmonella* infection of the reproductive organs i.e. ovaries or oviduct hence also called the transovarian route. In the transovarian route, the egg yolk membranes or albumen surrounding are directly contaminated [5]. Horizontal transmission is also called the trans-shell route in which *Salmonella* penetrates through the eggshell during or following oviposition [6]. A major cause of egg related *Salmonella* food poisoning cases and the most prevalent serovar in the layer industry across the world is *S.* Enteritidis; however, it is not endemic in Australian layer flocks [7]. In Australia and other parts of the world, *S.* Typhimurium is one of the most common serovars identified in egg borne salmonellosis cases [3], [36]. Horizontal transmission is the most common route by which salmonellae other than *S.* Enteritidis contaminate egg internal contents [8].

Important extrinsic factors such as the bacterial strain, temperature differential, moisture on the eggshell, the number of microorganisms in the inoculum and the storage conditions may affect eggshell penetration by *Salmonella* spp [5]. Intrinsic factors that may affect egg penetration include shell porosity, shell thickness and the extent of cuticle present on the shell [5]. There is also some evidence to suggest that eggshell translucency is associated with greater microbial penetration [9]. However, there is a lack of substantial literature on the relationship between translucency, eggshell ultrastructure and the penetration of bacteria.

Faeces, water, caging material, nesting material, insects, hands, broken eggs, blood, soil or dust on the egg belt are the most common sources of microbial contamination of the eggshell [10], [11]. Egg washing can reduce the microbial load on the eggshell surface [12] and thus may lower the rate of penetration of *Salmonella* across the eggshell and decrease the incidence of food poisoning. Egg washing is used to reduce eggshell contamination in many countries such as the United States, Australia and Japan [13]. However, some researchers claim that egg washing chemicals can damage the cuticle layer of the eggshell [14] which may result in moisture loss and deterioration of the internal quality of the egg. It is also possible that egg washing may favour the transmission of *Salmonella* across the eggshell particularly when the post-washing storage and drying conditions are substandard. As a result, there is currently a debate over the benefits of egg washing. Damage to the cuticle or alteration of the eggshell surface may change with different egg washing protocols [14] and may result in variation in the penetration of bacteria across the eggshell. There is a lack of information in this area in the Australian context.

In Australia, *S.* Typhimurium has been identified as the most prevalent serovar involved in cases of salmonellosis food poisoning [3]. Additionally, STm PTs (STm PT 9, STm PT 44, STm PT 135, STm PT 170 and STm PT 193) were frequently isolated or detected from egg products related to food poisoning cases in Australia [3], [15]. However, there is a lack of information available regarding how well these *S.* Typhimurium strains survive on the eggshell surface and penetrate the shell to contaminate the internal contents of the egg. A preliminary study suggested that eggshell ultrastructure may have an impact on eggshell penetration by *Salmonella* [16], but there is a lack of direct evidence.

The objectives of this study were to examine the effect of egg washing on the survival of various *S.* Typhimurium strains on the eggshell surface, to investigate the penetration ability of different *S.* Typhimurium strains, and to study the effect of egg washing on the bacterial penetration of the eggshell. The effect of egg washing on cuticle ultrastructure and the relationship between eggshell quality parameters and bacterial penetration were also investigated. Finally, the relationship between translucency and eggshell ultrastructure parameters and ease of bacterial penetration was studied. It was hypothesized that eggshell washing increases cuticle damage and also increases the rate of egg penetration by *S.* Typhimurium.

## Materials and Methods

Fresh and visibly clean table eggs were collected from hens less than 45 weeks old from a commercial Hyline layer farm located in South Australia near Adelaide. This study did not involve endangered or protected species. All table eggs used in this study were collected from cage front which did not involve handling of birds. Birds were not sacrificed for this study. Due to above reasons, animal ethics approval was not required. The farm was selected based on the willingness of the producers to participate in the study and specific permission was not required for egg collection. In the present experiment, five different *S.* Typhimurium strains were used, which had been initially characterised by reference laboratory from Australian layer farms, were obtained from the Australian *Salmonella* Reference Centre, Institute of Veterinary Medical Science (IMVS) in Adelaide, Australia. Each of these five strains was belonging to the different *S.* Typhimurium phage types (STm PT): Stain 1/group 1: STm PT 9; Strain 2/group 2: STm PT 44; Strain 3/group 3: STm PT 135; Strain 4/group 4: STm PT 170 and Strain 5/group 5: STm PT 193.

### Egg washing

The egg washing process used in this study involved the stages of pre-washing, washing with the aid of a surfactant, sanitizing and drying. A laboratory based washer which could hold 15 eggs in three rows of five rotating rollers was used for the physical mechanics of the egg washing. Washing was performed using a hydroxide and hypochlorite based solution at the concentration of 0.45% (v/v) which equates to a pH of ∼12 and ∼200 ppm hypochlorite in the working solution at 40°C. Washing was followed by a compatible sanitizer (at a concentration of 0.16% (v/v)) which equated to ∼200 ppm hypochlorite in the working solution at 32°C. Eggs were washed and sanitized for 46 and 22 seconds, respectively. The pressure of the spray was 3 psi without brushes. Eggs were left on the bench for 15 minutes to dry and used for further experiments.

### Inoculum preparation

Strains of *S.* Typhimurium stored at −80°C in 80% glycerol were plated on xylose lysine deoxycholate (XLD) agar (Oxoid, Australia) and incubated overnight at 37°C. Colonies were selected from XLD agar and resuspended in phosphate buffered saline (PBS) to match the turbidity equivalent with a 0.5 McFarland standard (BioMerieux, Australia). Enumeration of viable bacteria was performed by serial dilution and spread plating on XLD agar and incubation overnight at 37°C. Following enumeration, a 200 mL inoculum containing 10^3^ and 10^5^ colony forming units (CFU) per mL was prepared for each serovar. Agar filled eggs and whole eggs were immersed for 90 sec in one of three dilutions: PBS (control), ∼10^3^, and ∼10^5^ CFU/mL of *S.* Typhimurium.

### Whole egg penetration experiment to investigate the survival of *S.* Typhimurium on eggshell surface and in egg internal contents

The effects of egg washing on *S.* Typhimurium survival on the eggshell surface and penetration across the eggshell, as well as the survival of *S.* Typhimurium in the internal contents of the egg, were investigated using a ‘whole egg penetration’ approach. Ninety eggs were collected from HyLine Brown hens in early lay and were divided into two groups: washed (n = 30) and unwashed (n = 60). Washed eggs were divided into one control (PBS) and two treatment groups (10^3^ and 10^5^ CFU/mL) with 10 eggs each. All the washed eggs were incubated at 20°C after exposure to *S.* Typhimurium or the sham PBS treatment. Unwashed eggs were divided into two groups of 30 eggs. Group 1 was further divided into one control and two treatment groups (10^3^ and 10^5^ CFU/mL) of 10 eggs each. Eggs from group 1 were incubated at 20°C after exposure to *S.* Typhimurium or the sham PBS treatment. Group 2 was also divided into one control and two treatment groups (10^3^ and 10^5^ CFU/mL) of 10 eggs each. These unwashed eggs were incubated at 37°C. The reason that only unwashed eggs were incubated at 37°C is that washed eggs are not used for hatching purposes. Each egg was dipped into 70% ethanol for 30 sec to sterilize the outer shell and allowed to air dry in a biosafety cabinet for 10–15 min. Eggs were then immersed for 90 sec in 10^3^ CFU/mL or 10^5^ CFU/mL of *S.* Typhimurium. After inoculation, eggs were incubated at 20°C or 37°C for 21 days.

### Isolation of *S.* Typhimurium from eggshell surface and egg internal contents from whole egg penetration experiment

Eggshell surface samples and egg internal contents samples were processed separately by pooling two eggs together. After incubation, each egg was placed in a Whirl-Pak bag (Nasco, USA) containing 10 mL of buffered peptone water (BPW; Oxoid, Australia) and each egg was massaged for 1 min. A 100 µL aliquot of the mixture was spread plated onto XLD plates and incubated overnight at 37°C, and subsequently quantified.

To investigate the penetration and survival of *S.* Typhimurium in the internal contents of the egg, after the eggshell wash, eggs were dipped in 70% ethanol for 30 sec. Eggs were then aseptically opened, emptied into the Whirl-Pak bags and mixed. A 2 mL aliquot of the internal contents was transferred to 8 mL of BPW and 100 µL of this mixture was plated on XLD agar and incubated overnight at 37°C. Plates were then observed for *Salmonella* growth. Slopes of suspected *Salmonella* isolates were sent to the Institute of Medical and Veterinary Sciences (IMVS), Adelaide, Australia for confirmation.

### Polymerase Chain Reaction amplification for detection of *S.* Typhimurium

Detection of *S.* Typhimurium on the eggshell surface and in internal contents was achieved using a polymerase chain reaction (PCR) assay that targeted the *Salmonella*-specific *InvA* gene [17]. The pooled BPW (from two eggs), from the whole egg penetration experiment, for the eggshell surface and egg internal contents samples, was incubated overnight at 37°C. DNA was extracted and purified from these overnight incubated pooled BPW using the Wizard PlusMinipreps DNA purification system (Promega, Australia) as per manufacturer's instructions. Extracted DNA was suspended in nuclease free water, and stored at −20°C until further use. Each PCR reaction mixture contained 10×reaction buffer (Fisher Scientific, Australia), 1 mM MgCl_2_, 0.25 mM dNTPs, 5 µM forward *InvA* primer (5′- CTGGCGGTGGGTTTTGTTGTCTTCTCTATT-3′), 5 µM reverse *InvA* primer (5′-GTTTCTCCCCCTCTTCATGCGTTACCC-3′), 1.65 U *Taq* polymerase, and 10 ng DNA template, made up to 20 µL with nuclease free water. Samples were amplified using a Kyratech automated thermal cycler (Adelaide, Australia) with an initial denaturation step at 95°C for 5 min, followed by 30 cycles of amplification (denaturation at 95°C for 30 sec, annealing temperature 60°C for 30 sec and extension at 72°C for 1 min 30 sec), with a final extension step at 72°C for 5 min, followed by a holding temperature of 8°C. The separation of PCR products was done by 1.5% agarose gel electrophoresis in a Trisborate-EDTA (TBE) buffer. GelRed was used to visualize bands under ultra-violet light. The size of the PCR products was estimated using a 1 kb DNA ladder (Qiagen, Australia), with bands identified at 1062 bp.

The sensitivity of PCR was determined using serial dilutions. Cultures of *S.* Typhimurium stored at −80°C in 80% glycerol were plated on xylose lysine deoxycholate (XLD) agar (Oxoid, Australia) and incubated overnight at 37°C. Colonies were selected from the XLD agar and resuspended in BPW to match the turbidity of a 0.5 McFarland standard (BioMerieux, Australia). Enumeration of viable bacteria was performed by serial dilution and spread plating on XLD agar. DNA extraction and *Salmonella* specific PCR (as described above) was performed using serial dilutions of BPW. DNA extraction and *Salmonella* specific PCR was also performed from serially diluted BPW incubated overnight at 37°C.

### Agar method for assessment of the eggshell penetration with respected to washing, translucency and eggshell ultrastructural parameters

The effects of washing, translucency, and eggshell quality on the bacterial penetration of the eggshell were assessed by the ‘agar egg’ method as described previously [18]. Fresh eggs were obtained from the cage front of layers. All eggs were candled, scored for translucency, and allocated to two translucency groups based on candling score, until each group contained 32 eggs; where 1 = low translucency, and 2 = high translucency. For scoring translucency, a quantitative approach was used where a 1 cm^2^ area of eggshell was marked and the numbers of lighter coloured spots on the eggshell (as viewed over a light source) were counted. Eggshells with less than 10 spots/cm^2^ were considered to have low translucency. Eggs from each group were then allocated to washed and unwashed groups (n = 16 each) and subsequently allocated to inoculated (n = 10) and control groups (n = 6). Each egg was dipped into 70% ethanol for 30 sec for sterilization of the egg shell surface and aseptically air dried for 10–15 min.

The internal contents of each egg were removed using an 18 g needle (BD, Australia) at the blunt end of the egg. Eggs were also washed internally with sterile PBS (pH 7.2) to remove residual albumen. Eggs were then filled with XLD agar and sealed after the agar solidified. Agar-filled eggs from each treatment group (washed and unwashed) were immersed for 90 sec in 200 mL of approximately 10^5^ CFU/mL solution of *S.* Typhimurium. Eggs from the control groups (washed and unwashed) were immersed in sterile PBS for 90 sec. After inoculation, agar-filled eggs were incubated at 20°C for 21 days. After incubation, the eggs were aseptically opened and the penetration of *Salmonella* spp. was assessed by the blackening of the interior eggshell.

### Scanning electron microscopy (SEM)

A scanning electron microscope (SEM) (JCM-5000 NeoScope, JEOL, Japan) was used to score the ultrastructural features of the mammillary layer of the eggshell. A Dremel high speed rotary, model tool, 300 series was used to cut pieces of eggshell (approximately 1 cm^2^) from around the equator of all eggs. Eggshell pieces were soaked overnight in tap water. Shell membranes were then removed and eggshell pieces were air dried. Plasma etching of dried eggshell pieces was then performed using a BioRAD RF Plasma Barrel Etcher PT 7150 for 4 hours. Next, an air duster was used to remove ash particles. Each eggshell piece was mounted on a 9 mm diameter aluminium stub using I005Aqueous conductive silver liquid SEM adhesive (ProSciTech, Australia). The specimens were sputter coated in a Neocoater for 5 min, and viewed under the SEM (JCM-5000 NeoScope, JEOL, Japan) at various magnifications. Eggshell ultrastructural features of the mammillary layer were scored as per the following criteria: Cap size: similar = 1, variable = 2; Confluence: low = 1, high = 2; Cap quality: good = 1, poor = 2 ; Alignment: low = 1, high = 2, Type A bodies: low = 1, high = 2; Type B bodies: low = 1, high = 2; Argonite: Absent = 1, present = 2; Erosions: absent = 1, present = 2; Depression: absent = 1, present = 2.

Assessment of the cuticle was carried out using all the eggs in the control and treatment groups. Shell pieces of approximately 1 cm^2^ were cut out from around the equator of the eggshell using a Dremel tool, mounted on a 9 mm diameter aluminium stub, sputter coated and viewed under the SEM, as described above. Scoring of the cuticle was done according to Samiullah et al. [16] with following criteria: Cuticle score 1 = 90 to 100% cuticle cover, score 2 = 60 to 90% cuticle cover, score 3 = 20 to 60% cuticle cover, score 4 = 0 to 10% cuticle cover.

From all eggs, three pieces of shell with intact shell membranes were taken from around the equator of the egg to measure shell thickness. A custom-built gauge (based on a Mitutoyo Dial Comparator Gauge model 2019–10, Japan) was used to measure the shell thickness in micrometers.

### Statistical analysis of whole egg and agar penetration experiment

All statistical analyses were performed with the statistical software R version 2.15.0 [19]. Statistical analysis, for the whole egg penetration experiment, was conducted using the t-test (for eggshell surface samples- direct agar culture method) and Fisher's exact test (for internal contents samples- direct agar culture method). On the other hand, all the results from PCR were analysed using Fisher's exact test.

In the agar egg experiment, a logistic regression was used to assess the effects of washing, eggshell translucency and their interaction on eggshell penetration of inoculated eggs. Logistic regression was also used to explore the relationship between the overall egg shell structure and *S.* Typhimurium penetration. The overall eggshell structure was defined by the ultrastructural parameters of cap size, confluence, caps, alignment, the number of Type A and B bodies, the level of argonite, depression, erosion and shell thickness. An ordered logistic regression was used to model the effects of washing, translucency and treatment on the cuticle score of all 64 eggs. In addition, the relationship between translucency score and egg ultrastructure parameters was investigated using logistic regression. Models were assessed using Analysis of Variance (ANOVA) and based on a significance level of p<0.05, non-significant interactions were removed step-wise until only significant terms remained in the model. Model fit was assessed using standard diagnostic plots.

## Results

### Whole egg penetration experiment to study the survival of *S.* Typhimurium on eggshell surface and the contamination of egg internal contents

#### Survival of *S.* Typhimurium on the eggshell surface after 21 days of incubation

As the penetration of bacteria across the eggshell is dependent on the survival of bacteria on the eggshell, we compared the survival of *S.* Typhimurium strains on the eggshell surface of washed and unwashed eggs. Results indicated that there was no significant difference in the survival rate of *S.* Typhimurium strains on the eggshell surface of washed and unwashed eggs (Table 1). There was variation in the survival ability of different strains on the eggshell surface of washed and unwashed eggs. The effect of temperature (Table 2) on the survival of *S.* Typhimurium on the eggshell surface of unwashed eggs was studied using two different temperatures (20°C and 37°C). The survival rate of *S.* Typhimurium strain 2 (p = 0.02) and strain 5 (p = 0.0001) was significantly higher at 20°C (Table 2). For all *S.* Typhimurium strains, the overall trend indicated that a temperature of 20°C was more favourable for *S.* Typhimurium survival (Table 2). Using two different doses of inoculation (10^3^ and 10^5^ CFU/mL), the effect of dose on survival was studied and, as expected, results indicated that survival rate was higher in eggs inoculated with a 10^5^ CFU/mL dose (. However, a significant difference was observed only in the case of *S.* Typhimurium strain 4 (p = 0.02) (Table 3).

**Table 1:** Survival of *Salmonella* Typhimurium strains on eggshell surface after 21 days of incubation: Comparison between washed and unwashed eggs at 20°C.

*Salmonella* Typhimurium strain	Dose of infection (CFU/mL)	Washing status	Eggshell contamination after incubation (Log CFU/eggshell) Mean ± SE	p-value
*S.* Typhimurium strain 1	10^3^	Washed	3.09±0.82	0.68
		Unwashed	2.51±1.08	
	10^5^	Washed	4.09±0.27	0.34
		Unwashed	4.41±0.16	
*S.* Typhimurium strain 2	10^3^	Washed	2.69±1.09	0.54
		Unwashed	3.58±0.89	
	10^5^	Washed	4.49±0.01	0.37
		Unwashed	3.58±0.89	
*S.* Typhimurium strain 3	10^3^	Washed	2.23±0.92	0.33
		Unwashed	0.89±0.89	
	10^5^	Washed	3.42±0.92	0.34
		Unwashed	1.90±1.17	
*S.* Typhimurium strain 4	10^3^	Washed	0.89±0.89	0.62
		Unwashed	1.56±0.96	
	10^5^	Washed	4.34±0.21	0.27
		Unwashed	3.22±0.85	
*S.* Typhimurium strain 5	10^3^	Washed	1.67±1.04	0.59
		Unwashed	2.51±1.05	
	10^5^	Washed	2.12±0.88	0.06
		Unwashed	4.42±0.20	

**Table 2:** Survival of *Salmonella* Typhimurium strains on eggshell surface of unwashed eggs after 21 days of incubation: Comparison between 20°C and 37°C.

*Salmonella* Typhimurium strain	Dose of infection (CFU/mL)	Temperature (°C)	Eggshell contamination after incubation (Log CFU/eggshell) Mean ± SE	p-value
*S.* Typhimurium strain 1	10^3^	20	2.51±1.08	0.76
		37	3.04±1.24	
	10^5^	20	4.41±0.16	0.29
		37	4.69±0.18	
*S.* Typhimurium strain 2	10^3^	20	3.58±0.89	0.02
		37	ND	
	10^5^	20	3.58±0.89	0.02
		37	ND	
*S.* Typhimurium strain 3	10^3^	20	0.89±0.89	0.37
		37	ND	
	10^5^	20	1.90±1.17	0.18
		37	ND	
*S.* Typhimurium strain 4	10^3^	20	1.56±0.96	0.18
		37	ND	
	10^5^	20	3.22±0.85	0.05
		37	0.69±0.69	
*S.* Typhimurium strain 5	10^3^	20	2.51±1.05	0.07
		37	ND	
	10^5^	20	4.42±0.20	0.0001
		37	ND	

ND: not detected.

**Table 3:** Survival of *Salmonella* Typhimurium strains on the eggshell surface after 21 days of incubation: Comparison between different doses (10^3^ and 10^5^ CFU/mL) of infection.

*Salmonella* Typhimurium strain	Temperature (°C)	Washing status	Dose of infection (CFU/mL)	Eggshell contamination after incubation (Log CFU/eggshell) Mean ± SE	p-value
*S.* Typhimurium strain 1	20	Washed	10^3^	3.09±0.82	0.30
			10^5^	4.09±0.27	
		Unwashed	10^3^	2.51±1.08	0.15
			10^5^	4.41±0.16	
	37	Unwashed	10^3^	3.04±1.24	0.26
			10^5^	4.69±0.18	
*S.* Typhimurium strain 2	20	Washed	10^3^	2.69±1.09	0.17
			10^5^	4.49 ±.013	
		Unwashed	10^3^	3.58±0.89	1.00
			10^5^	3.58±0.89	
	37	Unwashed	10^3^	ND	NA
			10^5^	ND	
*S.* Typhimurium strain 3	20	Washed	10^3^	2.23±0.92	0.39
			10^5^	3.42±0.92	
		Unwashed	10^3^	0.89±0.89	0.51
			10^5^	1.90±1.17	
	37	Unwashed	10^3^	ND	NA
			10^5^	ND	
*S.* Typhimurium strain 4	20	Washed	10^3^	0.89±0.89	0.02
			10^5^	4.37±0.21	
		Unwashed	10^3^	1.56±0.96	0.23
			10^5^	3.22±0.85	
	37	Unwashed	10^3^	ND	0.37
			10^5^	0.69±0.69	
*S.* Typhimurium strain 5	20	Washed	10^3^	1.67±1.04	0.75
			10^5^	2.12±0.88	
		Unwashed	10^3^	2.59±1.05	0.11
			10^5^	4.47±0.20	
	37	Unwashed	10^3^	ND	NA
			10^5^	ND	

NA: Not applicable, ND: Not detected.

#### Penetration of eggs and contamination of internal contents by *S.* Typhimurium using direct agar culture method

Statistical analysis indicated that, at a dose of 10^5^ CFU/mL, the penetration of *S.* Typhimurium strain 2 into washed eggs was significantly higher (p = 0.04) compared to unwashed eggs (Table 4). In contrast, *S.* Typhimurium strain 3 penetration at 10^5^ CFU/mL was higher (p = 0.04) in unwashed eggs. For the other *S.* Typhimurium strains (1, 4 and 5), there was no significant difference in the *S.* Typhimurium penetration of washed and unwashed eggs. The effect of temperature on *S.* Typhimurium egg penetration was studied at 20°C and 37°C. *S.* Typhimurium strain 3 penetration (inoculated with 10^5^ CFU/mL) was significantly higher (p = 0.04) at 20°C (Data not shown). Temperature had no significant effect on the egg penetration of other *S.* Typhimurium strains. The effect of dose on egg penetration was also investigated using two different doses (10^3^ and 10^5^ CFU/mL). At 10^5^ CFU/mL, the penetration of strain 3 (at 20°C) was significantly higher (p = 0.04) compared to 10^3^ CFU/mL. However, for the remaining *S.* Typhimurium strains, there was no significant effect of dose on egg penetration (Data not shown).

**Table 4:** Whole egg penetration by different *Salmonella* Typhimurium strains: Comparison between washed and unwashed eggs at 20°C.

*Salmonella* Typhimurium strain	Dose of inoculation (CFU/mL)	Method of analysis	Group	Number of penetrated pools (of 2 eggs)	Number of non-penetrated pools (of 2 eggs)	p-value
*S.* Typhimurium strain 1	10^3^	Direct agar culture	Washed	1	4	1.00
			Unwashed	0	5	
		PCR	Washed	4	1	1.00
			Unwashed	5	0	
	10^5^	Direct agar culture	Washed	1	4	1.00
			Unwashed	1	4	
		PCR	Washed	3	2	1.00
			Unwashed	4	1	
*S.* Typhimurium strain 2	10^3^	Direct agar culture	Washed	2	3	1.00
			Unwashed	1	4	
		PCR	Washed	4	1	0.21
			Unwashed	1	4	
	10^5^	Direct agar culture	Washed	4	1	0.04
			Unwashed	0	5	
		PCR	Washed	5	0	0.04
			Unwashed	1	4	
*S.* Typhimurium strain 3	10^3^	Direct agar culture	Washed	0	5	1.00
			Unwashed	0	5	
		PCR	Washed	3	2	0.17
			Unwashed	0	5	
	10^5^	Direct agar culture	Washed	0	5	0.04
			Unwashed	4	1	
		PCR	Washed	4	1	1.00
			Unwashed	4	1	
*S.* Typhimurium strain 4	10^3^	Direct agar culture	Washed	0	5	1.00
			Unwashed	0	5	
		PCR	Washed	1	4	1.00
			Unwashed	0	5	
	10^5^	Direct agar culture	Washed	0	5	1.00
			Unwashed	1	4	
		PCR	Washed	4	1	0.52
			Unwashed	2	3	
*S.* Typhimurium strain 5	10^3^	Direct agar culture	Washed	1	4	1.00
			Unwashed	0	5	
		PCR	Washed	4	1	0.04
			Unwashed	0	5	
	10^5^	Direct agar culture	Washed	0	5	1.00
			Unwashed	1	4	
		PCR	Washed	5	0	0.04
			Unwashed	1	4	

#### PCR amplification for detection of *S.* Typhimurium

Results of PCR indicated that, in the case of all *S.* Typhimurium strains, there was no significant difference in the number of *Salmonella* positive eggshells from washed and unwashed eggs (Data not shown). When the effect of incubation temperature on the detection of *Salmonella* on the eggshell surface was studied, it was observed that *S.* Typhimurium strain 5 (10^5^ CFU/mL) detection was significantly higher (p = 0.04) at 20°C as compared to 37°C (Data not shown).

Results of PCR demonstrated that, *S.* Typhimurium strain 2 (inoculated with 10^5^ CFU/mL) and strain 5 (inoculated with 10^3^ and 10^5^ CFU/mL) penetrations were significantly higher (p<0.05) in washed eggs than unwashed eggs (Table 4). The effect of temperature on the *S.* Typhimurium egg penetration was studied and results suggested that strain 3 (inoculated with 10^5^ CFU/mL) penetration was significantly higher (p = 0.047) at 20°C compared to 37°C (Data not shown). When the effect of dose on egg penetration was studied, results showed that *S.* Typhimurium strain 3 (20°C) egg internal content contamination was significantly higher (p = 0.04) for eggs treated with 10^5^ CFU/mL as compared to 10^3^ CFU/mL. In other cases (except *S.* Typhimurium strain 1 at 20°C), egg penetration tended to be higher at an inoculation dose of 10^5^ CFU/mL as compared to 10^3^ CFU/mL but the difference was not significant (Data not shown).

#### Comparison of direct agar culture method and non-selective enrichment PCR

The limit of detection for the direct agar culture method and the combination of BPW enrichment without overnight incubation/PCR was 50 CFU/mL. The limit of detection of the combination of BPW enrichment with overnight incubation/PCR was 0.5 CFU. After 21 days of incubation, using the direct agar culture method, 51% of the eggshells were reported positive for *Salmonella* whereas, using PCR, a significantly higher (p = 0.0001) number of eggshells (82%) were observed to be *Salmonella* positive.

Using the direct agar culture method, it was observed that 16% of eggs were penetrated by *S.* Typhimurium but PCR results indicated that 49.33% egg internal contents were *Salmonella* positive. PCR detected a significantly higher (p = 0.0001) number of positive egg internal contents as compared to the direct agar culture method.

At 20°C and as per the direct agar culture method, 18% of washed eggs and 16% of unwashed eggs were penetrated by *S.* Typhimurium. However, PCR results indicated a very different scenario where a significantly higher (p = 0.0003) number of washed eggs (74%) were penetrated by *S.* Typhimurium as compared to unwashed eggs (36%), though it is not possible to determine whether these detections related to viable organisms.

### Agar egg penetration experiment for investigating the eggshell penetration with respected to washing, translucency and eggshell ultrastructural parameters

#### Relationship of washing and translucency with egg penetration by *S.* Typhimurium

A summary of the results for the number of washed and unwashed eggs which were penetrated for each *S.* Typhimurium strain and translucency score is given in Table 5. An analysis of only inoculated eggs indicated that *S.* Typhimurium penetrations were significantly higher for washed eggs than for unwashed eggs. All eggs –irrespective of their washing status or translucency score – were penetrated when inoculated with *S.* Typhimurium strain 2. For all *S.* Typhimurium strains, 80–100% of washed eggs were penetrated while only 40–70% of unwashed eggs were penetrated. In most cases, there was no significant difference in the number of penetrated eggs with low and high translucency scores, but translucency did have a significant relationship with the penetration of *S.* Typhimurium strain 5 (p = 0.02).

**Table 5:** Relationship of washing and translucency with agar egg penetration by *Salmonella* Typhimurium.

	Number of penetrated (non -penetrated) eggs					
	Low Translucency		High Translucency			
*Salmonella* Typhimurium strain	Unwashed	Washed	Unwashed	Washed	p-value (Relationship of washing with agar egg penetration)	p-value (Relationship of translucency with agar egg penetration)
*S.* Typhimurium strain 1	5 (5)	9 (1)	5 (5)	8 (2)	0.02	0.73
*S.* Typhimurium strain 2	10 (0)	10 (0)	10 (0)	10 (0)	1.00	1.00
*S.* Typhimurium strain 3	4 (6)	10 (0)	7 (3)	9 (1)	0.002	0.41
*S.* Typhimurium strain 4	4 (6)	9 (1)	4 (6)	9 (1)	0.0005	1.00
*S.* Typhimurium strain 5	8 (2)	10 (0)	4 (6)	8 (2)	0.02	0.02

#### Relationship of washing and translucency with egg cuticle score

**Figure 1:**
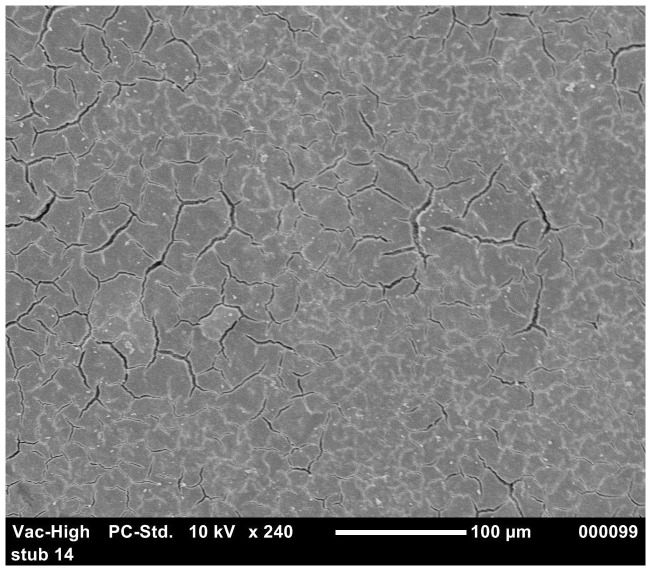
SEM image of good quality cuticle in unwashed eggs with no eggshell pores exposed.

**Figure 2:**
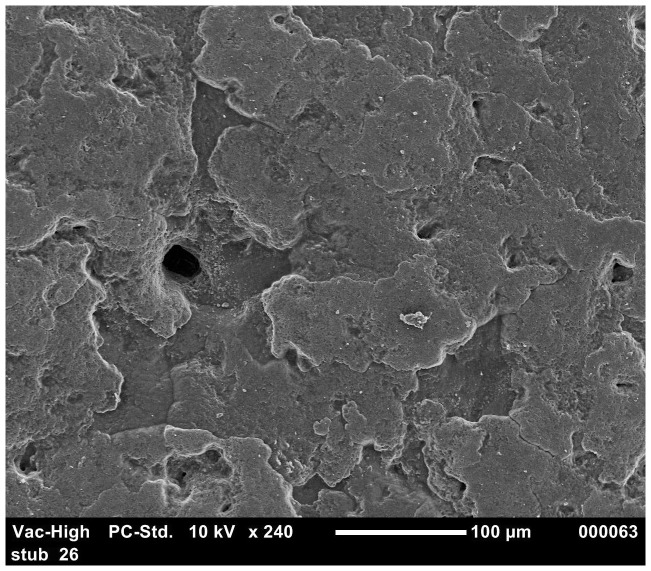
SEM image of damaged eggshell surface and exposed eggshell pore in washed eggs.

The results of the ordered logistic regression identified egg washing as having a significant effect on the cuticle score for all groups (Table 6). Fig. 1 and Fig. 2 show the good quality cuticle of an unwashed egg and the damaged cuticle/eggshell surface of a washed egg respectively. The interaction between washing and translucency was also significant for *S.* Typhimurium strain 3 (p = 0.002). Depending on the *S.* Typhimurium strain, 60–100% of washed eggs have a cuticle score of 3 or 4. This is compared to the cuticle scores of unwashed eggs being more evenly distributed between the four categories and 30–75% of unwashed eggs had cuticle scores of 3 or 4.

**Table 6:** Effect of egg washing on cuticle score.

*Salmonella* Typhimurium strain	Washing status	Number of eggs	Average cuticle score (Mean ± SE)	p-value
*S.* Typhimurium strain 1	Washed	32	3.60±0.09	0.0001
	Unwashed	32	2.75±0.15	
*S.* Typhimurium strain 2	Washed	32	3.25±0.15	0.05
	Unwashed	32	2.72±0.19	
*S.* Typhimurium strain 3	Washed	32	3.50±0.13	0.0001
	Unwashed	32	2.59±0.14	
*S.* Typhimurium strain 4	Washed	32	3.19±0.16	0.01
	Unwashed	32	2.59±0.16	
*S.* Typhimurium strain 5	Washed	32	2.81±0.16	0.01
	Unwashed	32	2.28±0.15	

#### Relationship between eggshell structure and eggshell penetration

**Figure 3:**
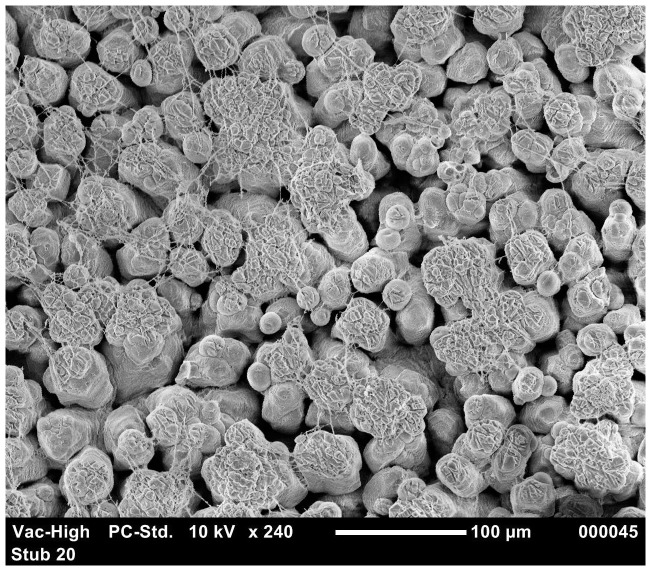
SEM image showing large number of Type B bodies in eggshell.

**Figure 4:**
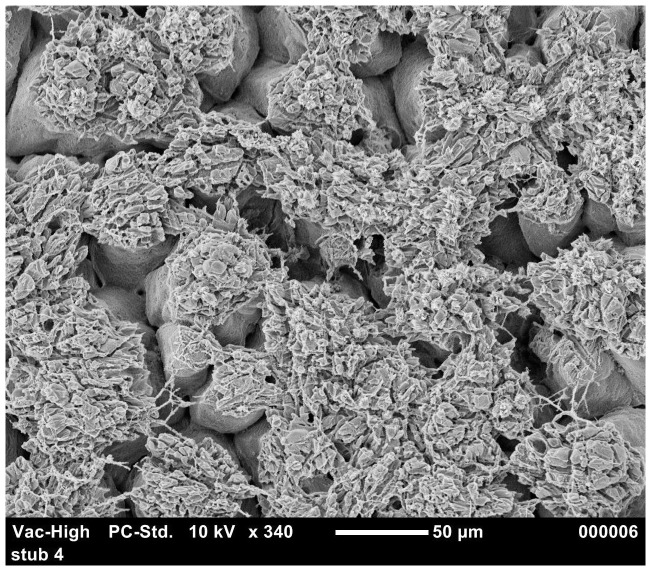
SEM image of good quality mamillary caps with better confluence.

**Figure 5:**
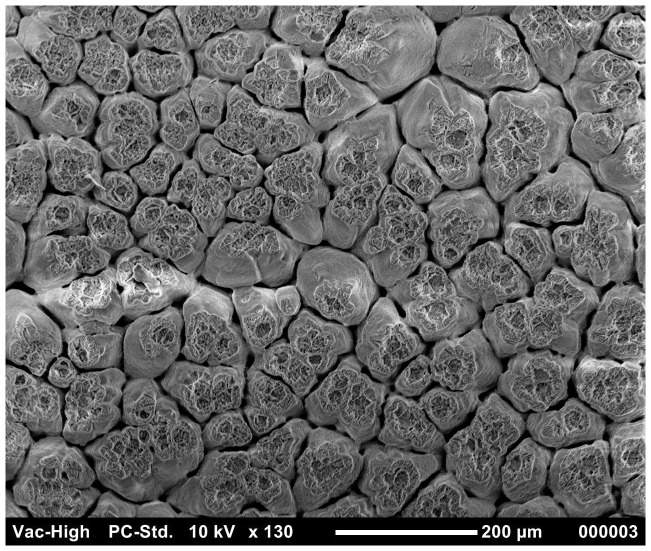
SEM image of extensive erosions throughout the eggshell.

**Figure 6:**
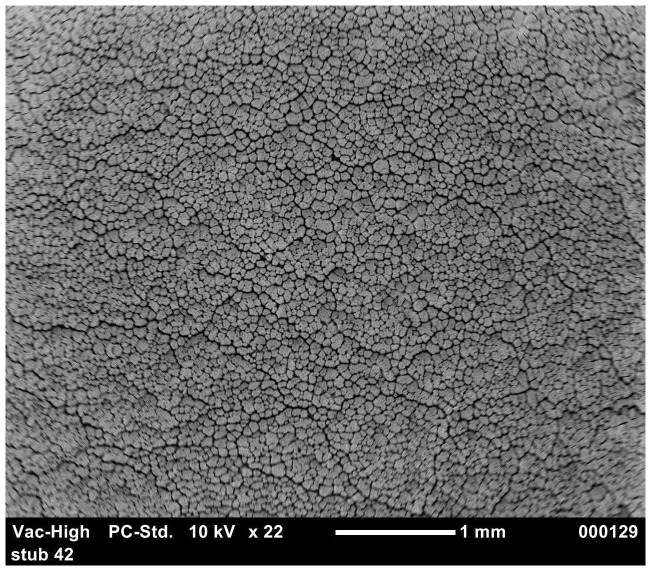
SEM image showing alignments in the mamillary layer.

To study the relationship between the eggshell structure and the susceptibility of the eggshell to penetration, we considered only the inoculated eggs and identified relationships between penetration and certain ultrastructure parameters, for each phage type. In Table 7 and 8, the details of statistical analysis are explained. Excluding variables that did not vary amongst inoculated eggs, the level of Type B bodies (Fig. 3) was a significant predictor of *S.* Typhimurium group 1 (strain 1) penetration (p = 0.01) (Table 8). In the case of *S.* Typhimurium group 3 (strain 3), cuticle (p = 0.001), confluence (p = 0.03) (Fig. 4) and cap quality (p = 0.0004) were significant predictors of eggshell penetration (Table 7). Results indicated that the levels of confluence (p = 0.01), alignment (p = 0.04) and erosion (p = 0.007) (Fig. 5) were significantly related to *S.* Typhimurium group 4 (strain 4) eggshell penetration (Table 7, 8). It was also observed that *S.* Typhimurium group 5 (strain 5) penetration was significantly related with eggshell ultrastructure parameters such as the level of confluence (p = 0.01), cap quality (p = 0.02), alignment (p = 0.03) (Fig. 6) and erosions (p = 0.009) (Table 7, 8). Statistical analysis also showed that, in most cases (except strain 3; p = 0.02), shell thickness was not related to eggshell penetration.

**Table 7:** Relationship between eggshell ultrastructural parameters and penetration by *Salmonella* Typhimurium.

	Number of penetrated (non-penetrated) eggs													
	Alignment			Cuticle score					Confluence			Cap quality		
*Salmonella* Typhimurium strain	Low	High	p-value	1	2	3	4	p-value	Low	High	p-value	Good	Poor	p-value
*S.* Typhimurium strain 1	4 (4)	23 (9)	0.44	1 (0)	4 (3)	6 (7)	16 (3)	0.09	21 (8)	6 (5)	0.51	9 (6)	18 (7)	0.97
*S.* Typhimurium strain 2	11 (0)	29 (0)	1.00	4 (0)	8 (0)	12 (0)	16 (0)	1.00	20 (0)	20 (0)	1.00	20 (0)	20 (0)	1.00
*S.* Typhimurium strain 3	11 (3)	19 (7)	0.83	0 (0)	5 (8)	9 (1)	16 (1)	<0.01	22 (6)	7 (4)	0.03	5 (3)	25 (7)	<0.01
*S.* Typhimurium strain 4	4 (7)	22 (7)	0.04	1 (1)	4 (7)	11 (4)	10 (2)	0.09	23 (7)	3 (7)	0.01	6 (11)	20 (3)	0.35
*S*. Typhimurium strain 5	9 (4)	21 (6)	0.03	6 (1)	5 (4)	13 (4)	6 (1)	0.46	29 (7)	1 (3)	0.01	2 (7)	28 (3)	0.002

**Table 8:** Relationship between eggshell ultrastructural parameters and penetration by *Salmonella* Typhimurium.

	Number of penetrated (non-penetrated) eggs														
	Type A bodies			Type B bodies			Erosion			Argonite			Depression		
*Salmonella* Typhimurium strain	Low	High	p-value	Low	High	p-value	Absent	Present	p-value	Absent	Present	p-value	Absent	Present	p-value
*S.* Typhimurium strain 1	25 (12)	2 (1)	0.39	13 (4)	14 (9)	0.007	15 (6)	12 (7)	0.07	27 (13)	0 (0)	NA	26 (13)	1 (0)	0.35
*S.* Typhimurium strain 2	37 (0)	3 (0)	1.00	24 (0)	16 (0)	1.00	25 (0)	15 (0)	1.00	31 (0)	9 (0)	1.00	39 (0)	1 (0)	1.00
*S.* Typhimurium strain 3	30 (10)	0 (0)	NA	3 (2)	27 (8)	0.64	13 (8)	17 (2)	0.09	30 (10)	0 (0)	NA	30 (10)	0 (0)	NA
*S.* Typhimurium strain 4	23 (14)	3 (0)	0.55	11 (7)	15 (7)	0.77	8 (6)	18 (8)	0.01	22 (14)	4 (0)	0.07	26 (14)	0 (0)	NA
*S.* Typhimurium strain 5	24 (10)	6 (0)	0.38	10 (5)	20 (5)	0.30	6 (2)	24 (8)	0.01	30 (10)	0 (0)	NA	30 (10)	0 (0)	NA

#### Relationship between eggshell ultrastructure parameters and translucency score

To investigate the relationship between translucency and eggshell ultrastructure parameters, the analysis identified different results for each *S.* Typhimurium strain (Data not shown). For *S.* Typhimurium group 1 (strain 1), none of the ultrastructure parameters were significantly related with translucency score and for group 4 (strain 4), there was a significant relationship only between cap size and translucency (p = 0.04). All eggs with high translucency (score 2) had a variable cap size (score 2) and approximately 9% of eggs with low translucency (score 1) had a similar cap size. For *S.* Typhimurium group 2 and 3 (strain 2 and strain 3), there were significant differences in erosion counts between eggs of differing translucency scores (p = 0.04 and 0.004 respectively). The analysis of shell thickness indicated that eggs with low translucency have significantly thicker shells on average compared to eggs with high translucency for *S.* Typhimurium group 2 (strain 2) (p = 0.02) and group 5 (strain 5) (p = 0.04). In contrast, eggs with high translucency and inoculated with *S.* Typhimurium group 3 (strain 3) have significantly thicker shells on average than eggs with low translucency (p = 0.02).

## Discussion

The penetration of bacteria across the eggshell is dependent on the survival of bacteria on the eggshell surface and egg storage conditions. Results from this study indicated that there was no significant difference in the survival rate of *S.* Typhimurium on the eggshell surface of washed and unwashed eggs.

For detecting *Salmonella*, PCR is a rapid, reliable and sensitive technique [17]. Hence, PCR was used in the present study to detect *Salmonella* on the eggshells as well as in egg internal contents in the whole egg penetration experiment. In the present experiment, the combination of Non-selective enrichment with overnight incubation/PCR was observed to be more sensitive as it was able to detect more positives. This result was not unexpected because PCR was performed on DNA extracted from bacterial cells in exponential phase. PCR results also indicated that, in the case of all *S.* Typhimurium strains, there was no significant difference in the number of *Salmonella* positive eggshells of washed and unwashed eggs. There is no information available in the literature to compare these findings. Direct agar method provided the actual counts of *Salmonella* whereas the results of PCR were qualitative. When the effect of temperature (20°C and 37°C) on the survival of *S.* Typhimurium on the eggshell surface was studied, the overall trend indicated that a temperature of 20°C is more favourable for *S.* Typhimurium survival on the eggshell surface at day 21 post inoculation (p.i.). These findings are in agreement with previous experiments which also reported better survival of *Salmonella* on the eggshell surface at lower temperatures [20], [21], [22], however, there were differences in the incubation temperatures and type of *Salmonella* serovar used in the previous study (*S.* Enteritidis).

For all *S.* Typhimurium strains, after 21 days incubation, 51% of the eggshells were positive for *S.* Typhimurium. These findings are in agreement with De Reu et al. [18] who found a high survival rate of *S.* Enteritidis on the eggshell surfaces after 21 days of incubation.

Results from whole egg penetration study also indicated that all *S.* Typhimurium strains used in the present study (at 20°C) were capable of penetrating the eggshells and surviving in the egg albumen which is considered to be a hostile environment for the survival of bacteria. Using the whole egg penetration approach, out of all the eggs tested, 16% of internal contents were observed to be positive for *S.* Typhimurium. This could be due to the antimicrobial properties of albumen. It was found that *S.* Typhimurium strain 2 penetration was significantly higher in washed eggs than unwashed eggs. For other *S.* Typhimurium strains (1, 4 and 5), there was no significant difference in the *S.* Typhimurium penetration of washed and unwashed eggs. Even though the results of the direct agar culture method showed that *S.* Typhimurium strain 3 penetration (at inoculation of 10^5^ CFU/mL) was higher in unwashed eggs, PCR results indicated that there was no significant difference. PCR results also indicated that *S.* Typhimurium strain 2 (10^5^ CFU/mL) and strain 5 (10^3^ and 10^5^ CFU/mL) egg penetration was significantly higher in washed eggs than unwashed eggs, which could be due to damage to the cuticle by egg washing chemicals.

The effect of temperature on *S.* Typhimurium penetration was studied at 20°C and 37°C. *S.* Typhimurium strain 3 penetrations (at inoculation of 10^5^ CFU/mL) was significantly higher at 20°C. For other *S.* Typhimurium strains, there was no significant effect of incubation temperature on egg penetration. Similar results were observed when samples were tested by PCR. Previous studies [23], [24] reported that, with the increase in temperature, the egg penetration of *Salmonella* was also increased. It is difficult to compare findings of these previous studies with the results of present experiment as in all these previous studies [14], [23], [24] eggs were incubated approximately at 4°C and 20°C whereas in the present experiment 20°C and 37°C temperatures were used to incubate eggs. The lower penetration at 37°C may be due to the reduced survival of *Salmonella* on the eggshell surface during incunation at this temperature.

The effect of dose on egg penetration was also investigated using two different doses (10^3^ and 10^5^ CFU/mL). At 10^5^ CFU/mL, the penetration of *S.* Typhimurium strain 3 (at 20°C) was significantly higher (p = 0.047) as compared to 10^3^ CFU/mL. PCR results also confirmed that the egg penetration by *S.* Typhimurium strain 3 was dependent on the dose of inoculation. These findings are consistent with a number of previous studies which indicated that the rate of contamination of eggs is directly proportional to the number of *Salmonella* in the culture used for infecting the eggs [6], [23], [24].

In agar penetration experiment, statistical analysis showed that *S.* Typhimurium strains penetration of washed eggs was significantly higher (p<0.005) than unwashed eggs. This may be due to the damage of cuticle by egg washing chemicals. To evaluate this further, the effects of washing on cuticle deposition was investigated using SEM. Results from the ordered logistic regression indicated that washed eggs had a significantly higher cuticle score (poor cuticle quality) as compared to unwashed eggs. These findings were not in agreement with a previous experiment where it was observed that egg washing had no significant effect on the quality of the cuticle [25]. The difference in the findings may be due to the variation in the age of laying hens and the difference in the protocol and chemicals of egg washing. In the present study, eggs were collected from younger hens (<45 weeks) in contrast to the previous experiment where eggs were collected from old laying hens (>54 weeks) [25]. It was previously observed that increasing age of laying hens has a negative impact on cuticle thickness [26], [27]. The variation in results of different experiments might result from the difference in the egg washing protocol [14]. In the present study, in case of *S.* Typhimurium strain 3, cuticle quality was observed as a significant predictor of *Salmonella* eggshell penetration. The mature cuticle closes the pores on the eggshell and protects the egg from the water and bacterial invasion [28] and the removal of cuticle or lower cuticle can result in increase in bacterial penetration [18], [29]. In the present study, using the agar approach, the relationship of translucency with the *S.* Typhimurium eggshell penetration was studied. Results indicated that, in most cases (except for strain 5), there was no significant relationship between translucency and eggshell penetration. However, Chousalkar et al. [9] reported a significant correlation between eggshell translucency and eggshell penetration by *S.* Infantis and *E. coli*. It is also essential to note that, in these two experiments, different bacterial strains were used to study eggshell penetration.

The SEM results were also analysed to study the relationship of eggshell quality parameters with the eggshell penetration. A higher incidences of alignment, erosions, poor cap quality, Type A mammillary bodies, Type B mammillary bodies may result in the weakening of the eggshell [30]. Small spherical bodies (Fig. 3) in the mamillary layer which may or may not have contact with membrane layer are known as Type B Bodies [31]. On other hand, the condition in which mamillary caps attach to each other is known confluence [31] (Fig. 4). Confluence is required for a stronger eggshell region [31]. Our results indicated that, for *S.* Typhimurium group 3 (strain 3) (p = 0.03), group 4 (strain 4) (p = 0.01) and group 5 (strain 5) (p = 0.01), eggshell penetration was negatively related to the level of confluence. The abrasion in mamillary layers are known as erosions (Fig. 5) which is believed to create the areas of weakness in eggshells [31]. Alignment in the eggshells is a situation where mammillae appeared to “line up” which may help to propagate a crack in eggshell [31] (fig. 6). Results also indicated that, for *S.* Typhimurium strain 4 and strain 5, eggshell penetration was positively related to a higher incidence of alignment and erosion. In case of *S.* Typhimurium group 3 and 5 (strain 3 and 5), eggshell penetration was negatively related to good cap quality. All these results are in agreement with the previous findings of Solomon [32] who reported that good mammillary caps and confluence can resist bacterial penetration whereas alignment, erosion and Type B bodies assist bacterial penetration. However, in the present study it was not clear as to why Type B bodies were negatively related to the incidence of *S.* Typhimurium group 1 (strain 1) eggshell penetration. Statistical analysis also showed that, in most cases, shell thickness was not related to the eggshell penetration. Similarly, a number of studies have observed that the shell thickness did not affect whole egg and agar egg penetration [16], [18], [33].

Our finding underlines the importance of proper storage and careful handling of eggs in the food industry and the domestic environment. Egg washing can reduce the level of *Enterobacteriaceae* (up to 4 log_10_) on the eggshell surface very efficiently [34] but, at the same time, results from agar penetration experiment indicated that the trans-shell penetration was higher in washed eggs than unwashed eggs. Hence, appropriate attention is essential to make sure eggs are kept at appropriate storage and drying conditions so that they will not come in contact with *Salmonella* after washing. In one study, swabs taken from multiple premises of grading machinery were reported positive for *Enterobacteriaceae* [34]; such situation could pose a higher risk of contamination of washed eggs. Hence, regular cleaning of the egg washing machine and grading equipment is essential to avoid recontamination of eggs once they are washed. Only in case of *S.* Typhimurium strain 2, 100% eggshell penetration was observed in washed and unwashed eggs. This suggests that *S.* Typhimurium strain 2 may have more capacity for trans-shell penetration compared to other *S.* Typhimurium strains used in this experiment. Luo et al. [35], using comparative genome analysis, showed that even highly similar *S.* Typhimurium strains could vary in their genome. In the current experiment, each *S.* Typhimurium strain belonged to different *S.* Typhimurium phage type. It is possible that strain variation was linked to phage type. However, further investigation using multiple isolates of same *S.* Typhimurium phage type is essential to confirm the variation in penetration ability of different phage types.
